# The impact of teachers’ innovative teaching behaviors on vocational college students’ learning engagement in the Chinese context: the mediating roles of AI trust and learning satisfaction

**DOI:** 10.3389/fpsyg.2026.1646199

**Published:** 2026-03-11

**Authors:** Tang Zhiwen, Ying Lin, Che Jing Shang

**Affiliations:** 1 Zhongshan Institute, University of Electronic Science and Technology of China, Zhongshan, China; 2 Student Affairs Department, Guangdong Women's Polytechnic, Guangzhou, China; 3 College of Education Science, Jiaying University, Meizhou, China

**Keywords:** artificial intelligence trust, innovative teaching behaviors, learning engagement, learning satisfaction, vocational university students

## Abstract

In the context of artificial intelligence (AI) integration in higher education—such as AI-supported learning platforms, automated feedback and grading systems, and generative AI tools like ChatGPT—teachers’ innovative teaching behaviors (ITB) are a key factor influencing students’ learning engagement (LE). While previous research has confirmed the positive effect of ITB on LE, the underlying psychological mechanisms, especially in AI-mediated environments, remain insufficiently understood. Guided by student engagement theory and social-cognitive perspectives on technology use, this study examined the parallel mediating roles of trust in AI (AIT) and learning satisfaction (LS) in the relationship between ITB and LE. A survey was conducted among 513 vocational university students in Guangdong, China—a region with advanced digital infrastructure and early AI adoption. The results showed that ITB significantly and positively predicted LE. Both AIT and LS acted as partial mediators in this relationship, suggesting that enhancing students’ satisfaction yields slightly stronger gains in engagement than focusing on AI trust alone. The findings indicate that teachers’ innovative practices enhance engagement both directly and indirectly by fostering learning satisfaction and cultivating calibrated, critical trust in AI—that is, confidence in AI’s usefulness combined with awareness of its limitations. This study is among the first to model AI trust and learning satisfaction as parallel mediators between innovative teaching behaviors and learning engagement, thereby enriching our understanding of how pedagogical innovation and students’ psychological perceptions jointly shape engagement in the AI era. These results imply that higher education should not only encourage innovative, AI-supported teaching designs but also deliberately nurture students’ emotional experiences (satisfaction) and their thoughtful, critical trust in AI tools to maximize learning engagement.

## Implications for practice

The research highlights that Learning Satisfaction (LS) is the most substantial mediator between innovative teaching and student engagement, accounting for nearly 30% of the total effect. This implies that “rehabilitating” the learning environment requires a focus on students’ emotional and subjective experiences. Institutions should implement feedback mechanisms to regularly assess student satisfaction with teaching methods, technologies, and content. The findings suggest that innovative practices such as personalized feedback, relevant real-world case studies, and interactive multimedia content should be actively promoted, as they enhance satisfaction and, consequently, deeper engagement.

## 1 Introduction

The integration of artificial intelligence (AI), particularly generative models like ChatGPT, into higher vocational education marks a paradigm shift in learning and skills training. In vocational universities and higher vocational colleges, AI is increasingly embedded in simulation systems, intelligent tutoring platforms, and workplace-oriented tools (e.g., AI-assisted design, diagnostics, or coding assistants), reshaping how students prepare for specific occupations. From Chat to Cheat (Lo and Chan, 2025) provides contextually proximate evidence that students’ AI use and trust are shaped not only by perceived opportunities and limitations, but also by peer/social contagion, policy awareness, and perceived detection effectiveness. This suggests that AI trust (AIT) is embedded in an institutional and normative ecology rather than functioning as a purely technological belief. Accordingly, in the present study AIT is theorized as a psychologically proximal mechanism that can be cultivated through classroom-level innovation, while its formation and downstream effects are plausibly conditioned by governance clarity, enforcement credibility, and peer climates. While the potential of AI to personalize learning and support skill acquisition is immense, its effectiveness is contingent upon a critical psychological factor: trust in AI (AIT) (Leite, 2025; Montag et al., 2024). The degree and nature of vocational university students’ trust in AI serve as a pivotal determinant of their academic and skills-based engagement.

When students hold a healthy, calibrated trust in AI, it can enhance their involvement across all three dimensions of engagement. For instance, by trusting AI tools to handle lower-order cognitive or routine tasks such as drafting practice reports, checking technical terminology, or simulating standard procedures, students can conserve mental resources. This reduction in cognitive load enables them to invest more deeply in higher-order thinking—analyzing complex cases, solving authentic work-based problems, and integrating theory with practice—thereby boosting cognitive engagement (Farrokhnia et al., 2024). At the same time, appropriate trust can increase behavioral engagement (e.g., more frequent practice with AI-enhanced training systems) and emotional engagement (e.g., interest, confidence) when students perceive AI as a reliable and supportive partner in their professional preparation (Jian et al., 2000). The traditional lecture-based model in higher education, including many vocational programs, has faced increasing scrutiny for its often passive nature and limited effectiveness in fostering deep learning, practical competence, and 21st-century skills (Freeman et al., 2014). In the vocational context, such approaches may be particularly insufficient for developing hands-on abilities, problem-solving skills, and workplace readiness. Consequently, there has been a growing emphasis on innovative teaching behaviors (ITB) that move beyond mere information transmission to create more dynamic, practice-oriented, and student-centered learning environments (Pan, 2024). ITB are a central focus of this study because they represent a proximal, malleable instructional lever through which vocational teachers can shape students’ psychological experiences—such as AIT and learning satisfaction (LS)—and, in turn, their learning engagement (LE). In other words, ITB operate at the instructional level, while AIT, LS, and LE operate at the level of students’ internal psychological drivers and outcomes, making ITB a theoretically and practically meaningful starting point for a mediation model in vocational higher education.

To enhance conceptual clarity, it is important to distinguish between instructional features and psychological drivers. Instructional features refer to observable aspects of teaching practice and course design, such as the use of AI-supported simulators in nursing or engineering, scenario-based training with generative AI, problem-based tasks rooted in real industry cases, flipped classrooms, and collaborative projects. Psychological drivers, by contrast, are students’ internal states and processes—including trust in AI, satisfaction with learning experiences, motivation, and self-regulation—that mediate how these instructional features translate into engagement and professional competence. In AI-mediated vocational settings, ITB function primarily as configurators of instructional features (e.g., how AI tools are integrated into workshops, labs, and internships), which then influence psychological drivers like AIT and LS.

Learning engagement is a multifaceted construct involving behavioral (e.g., attendance, practice time, participation in lab and workshop activities), emotional (e.g., interest, enjoyment, sense of relevance to future career), and cognitive (e.g., strategic learning, reflection, transfer of knowledge to workplace scenarios) dimensions (Christenson et al., 2012). In AI-mediated vocational contexts, ITB are expected to interact with these three dimensions in distinct yet interconnected ways. For example, AI-enhanced simulations and project-based tasks aligned with real occupational standards can increase behavioral engagement by prompting more frequent and sustained practice. Emotionally supportive and career-relevant AI-enhanced activities can foster emotional engagement, making vocational students feel more confident, curious, and committed to their professional pathway. Well-structured AI-assisted inquiry tasks—such as using ChatGPT to compare different technical solutions, generate alternative design options, or reflect on workplace scenarios—can promote cognitive engagement by encouraging deeper processing, metacognition, and the integration of theory with practical application. Thus, ITB are hypothesized to shape how vocational university students behaviorally, emotionally, and cognitively engage with AI-supported learning and training tasks.

This study therefore aims to elucidate the relationships between ITB, LS, AIT, and LE among vocational university students, positing that ITB positively influence AIT, LS, and LE, and that LS and AIT function as key psychological mechanisms linking ITB to different dimensions of engagement. Innovative teaching behaviors in vocational institutions encompass a range of practices that deviate from traditional, teacher-centered methods. These include the integration of technology (e.g., blended learning, gamification, virtual reality workshops, AI-based training systems), active learning strategies (e.g., problem-based learning around real workplace cases, ChatGPT-supported project work, flipped practical courses) (Dečman et al., 2025; Gu et al., 2025; Lee and Wu, 2025), authentic assessment methods (e.g., performance tasks aligned with industry standards), and approaches that foster creativity and critical inquiry (Tahir et al., 2024). The core of ITB lies in their student-centricity and practice-orientation, aiming to make learning more relevant to future careers, interactive, participatory, and tailored to diverse learner needs. Technology-enhanced vocational learning environments can offer personalized practice pathways and flexible access to resources, accommodating different learning paces, prior experience, and career goals. In this sense, ITB define how AI is integrated into vocational instruction and training (instructional features), which in turn shapes how students feel and think about their learning and future work (psychological drivers such as LS and AIT).

A substantial body of recent research suggests a strong positive correlation between teachers’ innovative teaching behaviors and students’ learning satisfaction (Gunness et al., 2023; Huang, 2025). In vocational settings, when educators adopt innovative, practice-relevant methods, students often perceive their learning environment as more stimulating, supportive, and closely aligned with workplace demands (Gomez Gomez, 2024). For example, active learning strategies that encourage collaboration on real-world projects can foster a sense of community and professional identity, which are vital components of satisfaction (Pitt and Winstone, 2023). The use of diverse teaching tools and techniques can also cater to a wider range of learning preferences and skill levels, preventing monotony and increasing perceived value. Furthermore, innovative approaches often involve providing timely and constructive feedback through novel channels—including AI-based feedback systems—which helps students understand their practical progress and areas for improvement, thereby enhancing their confidence and satisfaction with the learning process (Opsal et al., 2025; Pence, 2022). When teaching is perceived as directly relevant to students’ targeted occupations or career development—which ITB in vocational education explicitly strive to achieve through authentic tasks and industry-linked projects—satisfaction levels tend to rise (Wicaksono and Korom, 2023; Younes et al., 2022; Zengin et al., 2011). Thus, by creating more engaging, relevant, and professionally meaningful learning experiences, ITB directly contribute to higher levels of student satisfaction.

Innovative teaching behaviors are also instrumental in fostering greater learning engagement among vocational students. As noted, engagement is multidimensional, and innovative practices typically require students to be active participants rather than passive recipients of information. Problem-based learning around authentic workplace scenarios necessitates deep cognitive engagement as students grapple with complex, ill-defined problems (Ader et al., 2023; Urban and Urban, 2023). The integration of technology, such as interactive simulations, AI-driven skills trainers, or collaborative online platforms, can enhance both behavioral and cognitive engagement by providing immersive and realistic practice opportunities (Sailer and Homner, 2020). Emotionally, such environments can increase enjoyment, perceived relevance, and confidence, thereby sustaining engagement over time and strengthening students’ commitment to their chosen profession. Evaluating Teacher, AI, and Hybrid Feedback in English Language Learning (Lo et al., 2025) illustrates a concrete, design-based manifestation of innovative teaching behaviors (ITB)—teacher–AI hybrid feedback—and shows that curated, verification-oriented AI support can enhance learners’ motivation, quality, and performance. These outcomes align closely with learning satisfaction (LS) and learning engagement (LE), offering pedagogical support for our proposition that ITB can improve students’ learning experiences and engagement by shaping the quality and credibility of AI-supported learning processes. Building on this evidence, our practical implications emphasize hybrid, teacher-curated AI use (e.g., prompt scaffolding, verification routines, and feedback literacies) as actionable ways to raise LS and foster calibrated, critical trust in AI, thereby strengthening engagement.

Crucially, in AI-rich vocational environments, teacher intervention plays a central role in moderating and enhancing AIT. Instead of merely introducing an AI tool and instructing students on its basic functions, an innovative vocational teacher actively scaffolds the learning experience. This involves modeling critical interaction with AI—for example, demonstrating how to craft effective prompts for technical tasks, how to question the accuracy and safety of AI-generated solutions, and how to cross-check AI outputs with industry standards, manuals, or expert judgment (Miao et al., 2021; Vázquez-Madrigal et al., 2024). Through such guided practice, teachers shape students’ perceptions of AI’s competence, reliability, and limitations in real work contexts. This process does not simply increase trust; it refines it into a form of critical trust in AI—that is, confidence in AI’s usefulness combined with awareness of its biases, errors, and appropriate boundaries of use. Vocational students learn to trust AI conditionally and contextually, understanding when AI can be relied upon in simulations or decision support, and when human expertise and professional responsibility must take precedence. This calibrated, critical trust is expected to support more sustained and productive engagement with AI-supported learning and training tasks.

Accordingly, this study focuses on vocational university students and examines how teachers’ innovative teaching behaviors influence their learning engagement and trust in AI, and how learning satisfaction mediates these relationships, with the ultimate goal of providing guidance for enhancing the quality of higher vocational instruction in AI-mediated contexts.

## 2 Literature review and hypotheses development

### 2.1 Teachers’ innovative teaching behaviors and learning engagement

Teachers’ innovative teaching behaviors (ITB) refer to the adoption and implementation of novel pedagogical strategies, tools, and approaches that deviate from traditional, teacher-centered methods. These behaviors are characterized by a focus on student-centered learning, active participation, real-world problem-solving, and the thoughtful integration of technology (Xin et al., 2022). In higher vocational institutions, ITB are particularly important because they help align classroom learning with workplace demands and support students’ transition into specific occupations.

In the context of vocational universities, ITB typically include: Technology-enhanced instructional features, such as the use of digital tools, learning management systems (LMS), interactive whiteboards, simulations, virtual reality (VR), and artificial intelligence (AI) to create dynamic, practice-oriented learning experiences (Henderson et al., 2017). Active and collaborative learning designs, including think-pair-share, flipped classrooms, debates, case studies, and peer instruction, which require students to actively construct knowledge rather than passively receive information (El-Bassiouny and El-Naggar, 2023). Supportive classroom climate, in which students feel respected, valued, and safe to take intellectual and practical risks, which is crucial for emotional engagement (Tasiouli and Lyra, 2024). Differentiated and personalized instruction, where teaching methods, content, and assessments are tailored to the diverse learning paces, experiences, and career interests of vocational students (Iqbal et al., 2020). These instructional features are expected to influence a set of psychological drivers on the student side—such as motivation, self-efficacy, learning satisfaction, and trust in AI—which in turn shape students’ learning engagement. Student learning engagement (LE) is a multidimensional construct that extends beyond mere participation. It typically encompasses three core dimensions: behavioral, emotional, and cognitive engagement (Fredricks et al., 2004; Wang and Eccles, 2013). Behavioral engagement refers to students’ participation in academic and practice-based activities, including attendance, effort, persistence, and time-on-task in both classrooms and training workshops. Emotional engagement involves students’ affective reactions to teachers, peers, and the learning environment—such as interest, enthusiasm, enjoyment, and a sense of belonging to their vocational program. Cognitive engagement pertains to students’ psychological investment in learning, characterized by a willingness to exert mental effort, employ deep learning strategies, and develop self-regulation skills (Pohl, 2020; Wong et al., 2024). High levels of engagement across these dimensions are associated with improved critical thinking, problem-solving abilities, long-term retention of knowledge, and better preparation for the workplace (Pang, 2022; Shcheglova et al., 2019).

Existing research indicates several mechanisms through which ITB can promote each dimension of LE among vocational university students: Behavioral engagement: Active learning strategies inherently require students to be present and participate. Flipped classrooms, for instance, necessitate pre-class preparation and in-class application, directly impacting attendance, effort, and persistence (Rutkienė et al., 2022). The use of interactive technologies such as polling systems, collaborative online platforms, or AI-supported practice systems can also increase participation and time-on-task (Malekjafarian and Gordan, 2024). Cognitive engagement: Problem-based and project-based learning challenge students to think critically, analyze complex information, and apply theoretical knowledge to authentic vocational situations, thereby promoting deeper cognitive processing (Yu, 2024; Zheng et al., 2009). Technology-enhanced simulations or virtual labs allow students to experiment and learn from mistakes in a safe environment, encouraging sophisticated metacognitive strategies (Asare et al., 2023). Personalized learning paths can ensure that students are appropriately challenged, leading to sustained cognitive effort (Lin et al., 2024). Emotional engagement: Innovative methods that make learning more relevant, interactive, and enjoyable can significantly enhance students’ interest and motivation. For example, using real-world case studies or gamification can make abstract concepts more relatable and exciting, fostering a positive emotional connection to the subject matter and to the chosen occupation (Redondo-Rodríguez et al., 2022). A supportive, respectful classroom climate cultivated by ITB further strengthens emotional engagement by promoting belonging and confidence. Taken together, these findings suggest that ITB should be positively associated with vocational students’ learning engagement across all three dimensions. In line with this multidimensional view, the present study distinguishes behavioral, emotional, and cognitive engagement conceptually, while modeling LE as a higher-order construct in the structural model. Accordingly, we propose the following hypotheses about statistical associations in our cross-sectional data:

H1a: Teachers’ innovative teaching behaviors are positively associated with behavioral engagement among vocational university students.

H1b: Teachers’ innovative teaching behaviors are positively associated with emotional engagement among vocational university students.

H1c: Teachers’ innovative teaching behaviors are positively associated with cognitive engagement among vocational university students.

In addition, when we consider a composite measure of learning engagement, we expect:

H1d: Teachers’ innovative teaching behaviors are positively associated with overall learning engagement among vocational university students.

These hypotheses refer to expected patterns of association rather than causal effects, given the cross-sectional design of the study.

### 2.2 Learning satisfaction as a mediating variable

While a direct link between ITB and LE is intuitive and supported by prior research, the mechanisms underlying this relationship warrant further exploration, especially in vocational higher education. One key psychological driver is learning satisfaction (LS), defined as students’ subjective evaluation of their educational experiences, processes, and outcomes (Wiers-Jenssen et al., 2002). When vocational students perceive teaching methods as engaging, relevant to their future careers, and supportive of their learning needs—characteristics typically associated with ITB—their satisfaction is likely to increase, which in turn may fuel greater engagement (Djudin, 2018).

Learning satisfaction is a crucial indicator of the quality of educational experiences from the student perspective. It reflects students’ contentment with various aspects of their academic and training life, including teaching quality, curriculum relevance to industry, learning resources, assessment methods, and the overall learning environment. High LS has been linked to increased motivation, academic achievement, persistence, and positive word-of-mouth recommendations for the institution (Esteban and del Cerro, 2020). Among the many factors influencing LS, teacher-related variables—particularly teaching style, instructional quality, and the perceived innovativeness of teaching—consistently emerge as significant predictors.

In vocational universities, ITB are expected to enhance LS through several mechanisms. Innovative teaching methods are typically more interactive, participatory, and practice-oriented than traditional approaches (Freeman et al., 2014). For example, incorporating real-world problems (problem-based learning) can enhance cognitive engagement by requiring students to apply knowledge in authentic work contexts (Maftuh et al., 2023), which increases students’ perceptions of relevance and usefulness. The use of collaborative technologies and AI-supported platforms can foster behavioral engagement through peer interaction and repeated practice, while also supporting emotional engagement by creating a supportive learning community (Li et al., 2024). Personalized feedback facilitated by technology, engaging multimedia content, and active learning tasks that give students a sense of agency can significantly enhance their satisfaction with the teaching and learning process (Manalu, 2024). For vocational students in particular, when teaching is perceived as closely aligned with industry standards and future employment, satisfaction tends to rise. Therefore, ITB are expected to have both direct and indirect effects on LE. Directly, innovative teaching designs can increase behavioral, emotional, and cognitive engagement by making learning more active, meaningful, and professionally relevant. Indirectly, ITB can improve LS, which then motivates students to invest more effort, experience more positive emotions, and think more deeply about course content and practical skills. For instance, a well-designed flipped classroom in a vocational course (ITB) may lead students to feel more prepared and perceive in-class practice as more valuable (higher LS), which in turn encourages them to participate more actively and think more critically during simulations or lab sessions (higher LE; Han et al., 2021; Akçayır and Akçayır, 2018).

Based on this reasoning, we propose the following hypotheses:

H2: Teachers’ innovative teaching behaviors are positively associated with learning satisfaction among vocational university students.

H3a: Learning satisfaction mediates the positive association between teachers’ innovative teaching behaviors and behavioral engagement among vocational university students.

H3b: Learning satisfaction mediates the positive association between teachers’ innovative teaching behaviors and emotional engagement among vocational university students.

H3c: Learning satisfaction mediates the positive association between teachers’ innovative teaching behaviors and cognitive engagement among vocational university students.

**Figure 1:**
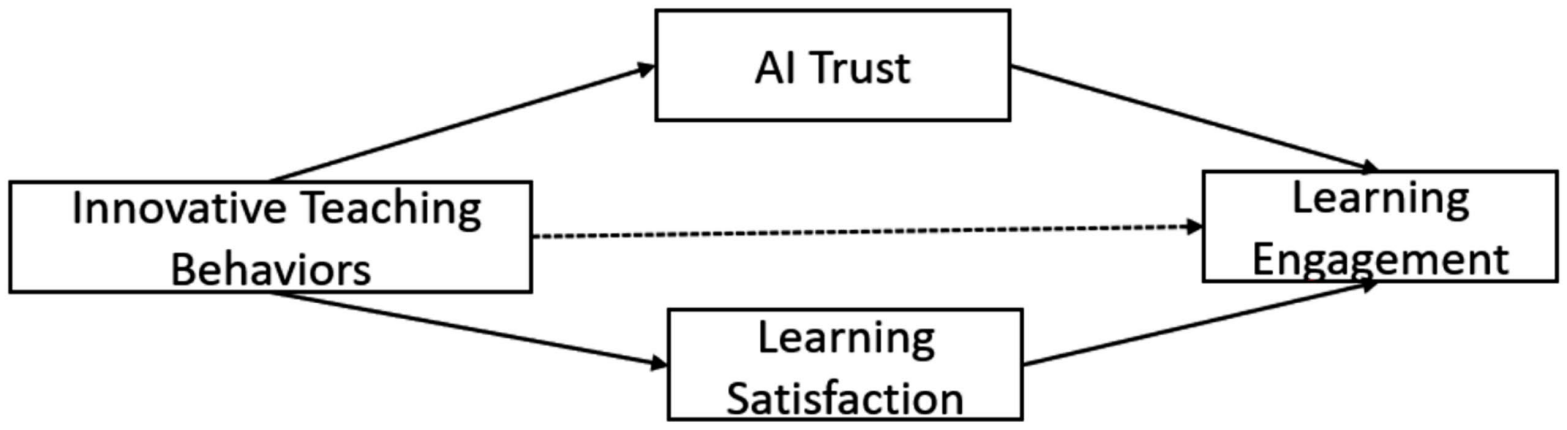
Theoretical model framework. Innovative teaching behaviors (ITB) by teachers directly enhance university students’ learning engagement (LE), ITB also indirectly foster LE through two parallel mediators: artificial intelligence trust (AIT) and learning satisfaction (LS).

Consistent with Figure 1 (Parallel multiple mediation model with AI Trust and Learning Satisfaction), these hypotheses refer to indirect (dashed) pathways from ITB to LE via LS, complementing the direct (solid) paths from ITB to the engagement dimensions in our cross-sectional model. We specified AIT and LS as parallel mediators rather than imposing a serial ordering (AIT → LS or LS → AIT) because, given the cross-sectional design, there is no strong theoretical or temporal basis to justify a directional causal sequence between AIT and LS; accordingly, modeling them in parallel provides a more defensible test of their simultaneous mediating roles.

## 3 Materials and methods

### 3.1 Participants

A total of 513 vocational university students were recruited by convenience sampling from several higher vocational institutions in Guangdong Province, China. Guangdong is a highly developed coastal province in southern China, with relatively advanced digital infrastructure and early adoption of AI in higher education, which makes it a suitable context for studying AI-mediated teaching and learning. The sampling frame consisted of full-time students enrolled in vocational bachelor and higher vocational diploma programs. Inclusion criteria were: (a) currently enrolled in one of the participating institutions; (b) able to complete an online questionnaire in Chinese; and (c) provided informed consent. Questionnaires with excessive missing data or patterned responses were excluded from analysis. Among the 513 valid respondents, 217 (42.3%) were male and 296 (57.2%) were female; Regarding academic year, 157 (30.6%) were first-year students, 167 (32.5%) second-year students, 189 (36.8%) third-year students, which sum to the total valid sample of 513.

Prior to analysis, we conducted data-quality screening to ensure the independence and credibility of responses. During this process, a number of questionnaires were identified as highly repetitive (i.e., duplicated cases with identical response patterns across a large proportion of items), suggesting non-independent submissions. These duplicated/repetitive cases were removed, together with questionnaires that showed excessive missing data or patterned responses (e.g., straight-lining). After these exclusions, the final analytic sample comprised *N* = 513.

This study was reviewed and approved by the Academic Ethics Committee of Zhongshan Institute, University of Electronic Science and Technology of China (2024011418). All procedures complied with the ethical standards of the institutional research committee and with the 1964 Helsinki Declaration and its later amendments.

### 3.2 Measures

All scales were originally developed in English and then translated into Chinese using a standard forward–backward translation procedure. Two bilingual experts independently translated the items into Chinese; discrepancies were resolved through discussion. A third bilingual expert, who was blind to the original instruments, back-translated the Chinese version into English. The back-translated items were compared with the originals to ensure semantic equivalence. Prior to the main survey, the questionnaire was piloted with a small sample of vocational students to check clarity and timing. Based on the pilot feedback, minor wording adjustments were made.

Confirmatory factor analyses (CFA) were conducted for all multi-item scales to verify the measurement structure in the present sample. Composite reliability (CR) and average variance extracted (AVE) indices were also computed to assess convergent and discriminant validity. Detailed psychometric results (factor loadings, CR, AVE, and HTMT values) are reported in Tables 1–3. Unless otherwise noted, all items were rated on a 5-point Likert-type scale. A 5-point format was chosen to balance measurement sensitivity with respondent burden and to be consistent with common practice in Chinese higher education research.

**Table 1:** Confirmatory factor analysis and reliability indices.

Variables	Item counts	Cronbach’s alpha	*ω*	AVE	CR
Innovative Teaching Behaviors	28	0.856	0.866	0.557	0.871
Artificial Intelligence Trust	12	0.810	0.831	0.524	0.862
Learning Satisfaction	21	0.823	0.835	0.747	0.801
Learning Engagement	16	0.852	0.861	0.652	0.830

**Table 2:** Model fit.

Variables	X^2^/df	df	SRMR	RMSEA	GFI	CFI	TLI
Innovative Teaching Behaviors	3.211	344	0.065	0.041	0.923	0.955	0.971
Artificial Intelligence Trust	3.010	101	0.071	0.031	0.958	0.962	0.956
Learning Satisfaction	3.560	189	0.074	0.043	0.965	0.956	0.967
Learning Engagement	4.212	54	0.079	0.038	0.967	0.966	0.978
Four Scales	3.432	2	0.077	0.036	0.974	0.954	0.974

**Table 3:** HTMT ratio matrix.

	ITB	AIT	LE	LS
ITB	1.000	[0.445, 0.598]	[0.478, 0.631]	[0.432, 0.587]
AIT	0.524	1.000	[0.575, 0.734]	[0.708, 0.807]
LE	0.555	0.652	1.000	[0.554, 0.709]
LS	0.510	0.764	0.634	1.000

Values below the diagonal are HTMT ratios; values above the diagonal are 95% BCa bootstrap CIs based on 5,000 resamples. Discriminant validity was assessed using the criterion HTMT < 0.85.

#### 3.2.1 Innovative teaching behaviors (ITB)

Students’ perceptions of their teachers’ innovative teaching behaviors were measured using a student-perceived version of the Creative Teaching Behavior Scale originally developed by Kay and revised by Jinghuan et al. (2008). The original teacher self-evaluation items were adapted to reflect students’ perspectives (e.g., “I encourage students to use multiple methods to solve problems”) were rephrased as “My teachers encourage me to use multiple methods to solve problems.” This adaptation is consistent with prior studies that have used student ratings to assess teachers’ innovative practices in classroom contexts.

The adapted scale consists of 28 items covering four dimensions: Guidance on learning methods, Motivational stimulation, Evaluation of viewpoints, Encouragement of flexibility.

Students responded on a 5-point Likert scale (1 = “never” to 5 = “always”). Higher scores indicate a higher level of perceived innovative teaching behaviors by their teachers. In this study, Cronbach’s alpha for the overall ITB scale was 0.856. CFA results and validity indices (CR, AVE) are reported in Tables 1, 2.

#### 3.2.2 Learning engagement (LE)

Learning engagement was assessed using the Utrecht Work Engagement Scale for Students (UWES-S) developed by Schaufeli et al. (2002), adapted into Chinese and modified by Laitan et al. (2008). The scale encompasses three dimensions:

Vigor (e.g., “When I study, I feel bursting with energy”),

Dedication (e.g., “I am enthusiastic about my studies”), and

Absorption (e.g., “I am immersed in my studies”).

In the present study, 16 items were used. Students rated each item on a 5-point scale (1 = “never” to 5 = “always/every day”). Higher scores indicate higher levels of learning engagement. Cronbach’s alpha for the total LE scale was 0.852.

Given the three-dimensional conceptualization of engagement. The fit indices and factor loadings for the final retained model are reported in Table 1. In the structural analyses, LE was modeled as a latent construct, with its three dimensions as first-order factors.

#### 3.2.3 Learning satisfaction (LS)

Learning satisfaction was measured with the scale developed by Wang and Yang (2016). This instrument was designed for Chinese higher education and is suitable for vocational contexts. It consists of 21 items across four dimensions:

Teaching attitude (e.g., “I am satisfied with my teachers’ enthusiasm and responsibility”),

Teaching content (e.g., “The course content is closely related to my future career”),

Teaching methods (e.g., “The teaching methods used in class are diverse and effective”), and

Teaching effectiveness and cultivation of innovative ability (e.g., “The course improves my ability to think creatively and solve problems”).

Students responded on a 5-point Likert scale (1 = “very dissatisfied” to 5 = “very satisfied”). Higher scores reflect higher learning satisfaction. In this study, Cronbach’s alpha for the overall LS scale was 0.823. CFA and validity statistics (CR, AVE) are summarized in Table 1.

#### 3.2.4 Artificial intelligence trust (AIT)

Trust in AI was assessed using a Chinese version of the trust in automation scale originally developed by Jian et al. (2000). Following a translation and back-translation process, the items were adapted to the educational AI context (e.g., “I can trust this AI tool” and “This AI system is dependable in supporting my learning”). The final scale includes 12 items.

Students rated each item on a 5-point Likert scale (1 = “strongly disagree” to 5 = “strongly agree”). Higher scores indicate higher levels of trust in AI used in their learning. In the present sample, Cronbach’s alpha for the AIT scale was 0.810. CFA results and validity indices (CR, AVE) are reported in Table 1.

### 3.3 Procedure

Data were collected using the online platform Questionnaire Star (Wenjuanxing), which is widely used for survey research in China. Course instructors and program coordinators in the participating vocational universities distributed the survey link to eligible students via institutional learning management systems and class communication groups. Before accessing the questionnaire, students were presented with an online information sheet describing the purpose of the study, procedures, voluntary nature of participation, potential risks and benefits, and data-protection measures. Students who agreed to participate clicked an “I agree” button to provide electronic informed consent. Participation was anonymous; no identifying personal information (e.g., student ID, name, phone number) was collected. Data were stored on password-protected servers and were accessible only to the research team. After providing consent, participants first completed demographic questions (e.g., gender, age, academic year, major), followed by the Innovative Teaching Behaviors scale, the Artificial Intelligence Trust scale, the Learning Engagement scale, and the Learning Satisfaction scale (Table 3).

### 3.4 Data analysis

Confirmatory factor analyses (CFAs) were conducted in AMOS to examine the measurement properties of the four constructs (ITB, AIT, LS, and LE). For hypothesis testing, we did not estimate a full structural equation model (SEM) with global model-fit indices. Instead, composite scores were computed for each construct and the proposed parallel mediation model was tested using ordinary least squares (OLS) regression–based mediation in PROCESS (Model 4; Hayes), with ITB as the predictor, AIT and LS as parallel mediators, and LE as the outcome. Indirect effects were evaluated using bias-corrected bootstrapping with 5,000 resamples, and effects are reported as unstandardized coefficients (B) with standard errors and 95% confidence intervals. All analyses were conducted using the final analytic sample *N* = 513.

## 4 Results

### 4.1 Reliability and validity tests

In the present study, we did not conduct an additional latent method factor (LMF) test for common method variance. Given the large number of items and the multidimensional structure of the measurement model, adding an LMF would substantially increase model complexity and could lead to estimation problems (e.g., nonconvergence or improper solutions), thereby reducing interpretability. Instead, we implemented procedural remedies (e.g., anonymous and voluntary participation, emphasizing that there were no right or wrong answers, and minimizing evaluation pressure) and assessed construct distinctiveness statistically. Specifically, the Harman’s single-factor test indicated that the first factor accounted for 35.6% of the variance, suggesting that a single general factor did not dominate the covariance among items. In addition, the CFA results and HTMT ratios supported satisfactory discriminant validity among the focal constructs. Nevertheless, because all variables were measured via self-report in a single survey wave, common method variance cannot be completely ruled out and should be considered when interpreting the findings. Future research may apply an LMF approach under a more parsimonious measurement structure, or adopt multi-source and/or time-separated designs to further mitigate and test for method effects.

The present study followed the guidelines of Fornell and Larcker (1981) to assess the reliability and validity of the scale.

HTMT ratios (off-diagonal), with 95% bootstrap Cis (*n* = 5,000 samples, above diagonal). All values <0.85, supporting discriminant validity (Table 4).

**Table 4:** Correlation analysis between the various variables.

	Innovative teaching behaviors	Artificial intelligence trust	Learning satisfaction	Learning engagement
Innovative teaching behaviors	1			
Artificial intelligence trust	0.520***	1		
Learning satisfaction	0.500***	0.533***	1	
Learning engagement	0.510***	0.505***	0.632***	1

***p < 0.001.

### 4.2 Correlation analysis of innovative teaching behaviors, artificial intelligence trust, learning engagement, and learning satisfaction

#### 4.2.1 Mediation model

Table 5 reports the bootstrap-based mediation tests (5,000 resamples) for the proposed model. Innovative Teaching Behaviors (ITB) significantly predicted Artificial Intelligence Trust (AIT) [(*R*^2^ = 0.250), (*F* = 170.016), (*B* = 0.676), (*t* = 13.039), (*p* < 0.001)] and Learning Satisfaction (LS) [(*R*^2^ = 0.260), (*F* = 179.347), (*B* = 0.412), (*t* = 13.392), (*p* < 0.001)]. When predicting Learning Engagement (LE), the overall model was significant [(*R*^2^ = 0.462), (*F* = 145.605), (*p* < 0.001)], and ITB remained a significant predictor [(*B* = 0.245), (*t* = 6.475), (*p* < 0.001)]. In addition, both mediators showed significant positive effects on LE, with AIT [(*B* = 0.173), (*t* = 4.009), (*p* < 0.001)] and LS [(*B* = 0.367), (*t* = 5.032), (*p* < 0.001)] contributing uniquely to explaining LE. Taken together, these results are consistent with a partial mediation pattern in which ITB influences LE both directly and indirectly through AIT and LS.

**Table 5:** Mediation model tests for ITB (bootstrap = 5,000).

Outcome Variable	Predictor	*R* ^2^	*F*	*B*	*t*
Artificial intelligence trust (AIT)		0.250	170.016***		
	Constant			1.544	9.836***
	ITB			0.676	13.039***
Learning satisfaction (LS)		0.260	179.347***		
	Constant			1.564	16.799***
	ITB			0.412	13.392***
Learning engagement (LE)		0.462	145.605***		
	Constant			0.587	4.773***
	ITB			0.245	6.475***
	AIT			0.173	4.009***
	LS			0.367	5.032***

*N* = 513. ITB = innovative teaching behaviors; AIT = artificial intelligence trust; LS = learning satisfaction; LE = learning engagement. ***p < 0.001.

The bootstrap mediation results (5,000 resamples) show that ITB has a significant total effect on learning engagement (LE) ((*c* = 0.514), 95% CI ([0.441,0.587])). After including the mediators, the direct effect of ITB on LE remains significant ((*c*’ = 0.245), 95% CI ([0.171,0.319])), indicating partial mediation. The total indirect effect is also significant ((0.269), 95% CI ([0.215,0.326])). Specifically, ITB influences LE through AI Trust ((0.116), 95% CI ([0.047,0.188])) and through Learning Satisfaction ((0.153), 95% CI ([0.085,0.229])). Because none of the bootstrapped confidence intervals include zero, both mediating pathways are supported, with the Learning Satisfaction pathway showing the larger indirect effect than AI Trust.

## 5 Discussion

### 5.1 Direct impact of innovative teaching behaviors on learning engagement: validating and extending theory

This study found that ITB has a significant positive predictive effect on overall LE (β=0.5134,p<0.001) , a result highly consistent with the research conclusions of Wang and Eccles (2013) and Xin et al. (2022). The strong correlation between ITB and LE in this study (r=0.518) empirically supports the theoretical claim that innovative teaching is closely linked to student engagement. It is important to note, however, that our empirical model treated learning engagement as a single latent construct; the specific process mechanisms described below are literature-based and were not directly tested as separate mediational paths in our data.

Drawing on prior research, at least three mechanisms have been theorized through which ITB may enhance engagement. At the behavioral engagement level, previous work suggests that innovative teaching methods—such as flipped classrooms—can significantly improve students’ classroom participation (Rutkienė et al., 2022). When teachers employ interactive polling systems or collaborative online platforms (Malekjafarian and Gordan, 2024), students’ task engagement time is theorized to increase. Our finding of a robust association between ITB and overall LE is consistent with, but does not by itself prove, this behavioral mechanism (Table 6).

**Table 6:** Mediation effect of artificial intelligence trust and learning satisfaction.

Effect type and path	B	SE	Boot BI lower limit	Boot BI upper limit
Total and direct effects				
Total effect of ITB on LE (c)	0.514	0.037	0.441	0.587
Direct effect of ITB on LE (c′)	0.245	0.038	0.171	0.319
Indirect effects of ITB on LE				
Total indirect effect	0.269	0.028	0.215	0.326
Via AI Trust (ITB → AIT → LE)	0.116	0.036	0.047	0.188
Via learning satisfaction (ITB → LS → LE)	0.153	0.037	0.085	0.229

ITB = innovative teaching behaviors; LE = learning engagement; AIT = AI Trust; LS = learning satisfaction. Coefficients are unstandardized. Total effect (*c*) and direct effect (*c*′) confidence intervals are based on the ordinary least squares estimates reported by PROCESS Model 4. Indirect effects and their confidence intervals are based on 5,000 bootstrap samples (bias-corrected 95% CIs). All confidence intervals that do not include zero indicate statistically significant effects at *p* < 0.05.

At the cognitive engagement level, studies by Yu (2024) and Zheng et al. (2009) indicate that problem-based and project-based learning promote deep cognitive processing. In particular, when teachers introduce AI-assisted simulation experiments or virtual laboratories (Asare et al., 2023), students can engage in trial-and-error learning in safe environments and develop more complex metacognitive strategies. Our data are compatible with this account insofar as higher ITB predicts higher composite engagement, but we did not include separate indicators of specific cognitive strategies in the structural model, so these mechanisms remain inferential rather than directly tested.

At the emotional engagement level, prior research has emphasized the role of gamified teaching in enhancing students’ interest and enjoyment (Redondo-Rodríguez et al., 2022). By using real case studies and interactive activities, innovative teaching methods can make abstract concepts more relevant and attractive, thereby fostering positive emotional connections to the subject. Again, our findings provide empirical support for a strong overall link between ITB and engagement, while the tripartite mechanisms across behavioral, cognitive, and emotional engagement are grounded in the literature rather than separately estimated in our data.

### 5.2 Mediating role of learning satisfaction: from theory to empirical evidence

This study found that LS plays an important mediating role between ITB and LE, with the mediation effect accounting for 29.84% of the total effect. This finding not only validates Hypothesis 3 proposed in the literature review but also provides empirical evidence for the theoretical mechanism summarized there. Specifically, the path analysis shows that the indirect pathway ITB → LS → LE is significant, consistent with the research conclusions of Han et al. (2021).

Theoretically, Djudin (2018) argued that when students perceive teaching methods as attractive, relevant, and responsive to their learning needs—features that characterize innovative teaching behaviors—their satisfaction increases, which in turn drives deeper engagement. Our empirical results directly support this core mechanism at the latent-variable level: ITB significantly predicts LS, and LS significantly predicts LE, with a non-trivial proportion of the ITB–LE relation transmitted through satisfaction. At a more fine-grained level, prior studies emphasize the role of personalized feedback, multimedia content, and active learning tasks in enhancing satisfaction (Manalu, 2024). While our measures did not disentangle the unique contributions of each instructional feature, the significant mediating effect of LS suggests that students’ holistic evaluative experience of teaching is an important psychological bridge between ITB and engagement.

### 5.3 Unique mediating role of artificial intelligence trust: theoretical innovation and practical implications

A significant contribution of this study is revealing the mediating role of AIT in the ITB–LE relationship, with AIT accounting for 22.17% of the total effect. At the empirical level, our path model shows that the chain ITB → AIT → LE is significant. This provides direct support for the theoretical claim by Zehner and Hahnel (2023) that students’ trust in AI systems’ fairness, accuracy, and educational value is crucial for their active participation in AI-supported learning.

Our teaching-focused framing of AIT also helps clarify the mechanistic interpretation of this mediation. When teachers use innovative methods to demonstrate AI tools—for example, explicitly modeling how to design effective prompts, how to critically question AI-generated responses, and how to cross-verify outputs with authoritative sources, as promoted by Miao et al. (2021) and Vázquez-Madrigal et al. (2024)—students are more likely to perceive AI as both useful and controllable. Our data show that higher ITB is associated with higher AIT, and that higher AIT in turn predicts greater overall engagement, but the specific cognitive, emotional, and behavioral pathways through which trust operates are inferred from theory rather than decomposed in the model.

With regard to the three dimensions of engagement, prior literature suggests that AIT may operate somewhat differently across behavioral, emotional, and cognitive facets. Conceptually, students who trust AI are more likely to (a) behaviourally persist in using AI tools rather than abandoning them after initial difficulty, (b) emotionally experience less anxiety and more curiosity when interacting with AI, and (c) cognitively invest effort in exploring, evaluating, and integrating AI-generated information into their own thinking. In this study, however, LE was modeled as a single latent construct that integrates behavioral, emotional, and cognitive items. As a result, we could not statistically estimate separate paths from AIT to each engagement facet, and our findings speak to AIT’s relationship with overall learning engagement rather than its differential effects. Future research should explicitly model three distinct engagement factors to test whether AIT is more strongly tied to emotional–cognitive engagement than to purely behavioral participation.

Beyond individual-level processes, our findings also connect to institutional readiness and policy. Furthermore, Chan and Hu (2023) warned that lack of trust leads to student resistance, disengagement, or merely superficial use of powerful educational tools. Our results suggest that “conditional trust” cultivated through innovative teaching behaviors—where students understand both the appropriate contexts for AI application and the need for critical vigilance—can mitigate these risks. Effective AI integration in higher education therefore requires targeted institutional policies that enhance technology readiness, promote a culture of peer support, and address educators’ AI-related concerns, so that classroom-level trust-building efforts by teachers are reinforced rather than undermined by the broader environment.

Finally, the present findings highlight that trust in AI should not be equated with uncritical acceptance. This study underscores the importance of critical trust formed through teacher guidance, rather than blind trust, as a key factor in promoting deep learning engagement. Proactive, ethically grounded integration of AI—guided by institutional oversight, curriculum reform, and equity safeguards—is essential to ensure its educational benefits while preserving integrity and humanistic values In this sense, teacher practices that foster reflective, well-informed trust function as a micro-level expression of broader ethical governance in AI integration.

### 5.4 Research limitations and future directions

Although this study provides valuable findings, several limitations should be acknowledged. First, as described in the literature review, the sample comes from universities in the Guangdong region, and regional characteristics may affect the generalizability of the results. Future research should expand to different regions and types of institutions to test the robustness of these patterns.

Second, this study relied exclusively on self-report measures, which may increase the risk of social desirability bias and common method variance. Although we collected the data anonymously, assured participants of confidentiality, and employed well-validated scales, these procedures cannot completely rule out the possibility that respondents answered in a socially desirable manner. Future research could address this limitation by combining self-reports with additional data sources—such as classroom observations, learning analytics, behavioral indicators of system use, or peer and instructor ratings—in order to triangulate the key constructs and further enhance the robustness of the findings.

Third, the study did not distinguish the differential impacts of different types of AI tools (such as large language models like ChatGPT, AI recommendation systems, or automated scoring systems) on trust and engagement. Different tools may evoke distinct patterns of perceived usefulness, fairness, and risk, which could in turn shape AIT and engagement in different ways. Subsequent studies should therefore examine specific AI applications and compare their effects on behavioral, emotional, and cognitive engagement.

Finally, our structural models treated learning engagement as a single latent construct. Although this approach aligns with many prior studies, it limited our ability to test whether ITB and AIT relate differentially to behavioral, emotional, and cognitive engagement. Future research should employ multidimensional engagement models and longitudinal or experimental designs to more rigorously identify the causal processes through which innovative teaching behaviors and AI trust jointly shape students’ learning experiences.

## 6 Conclusion

This study supports and extends the theoretical framework proposed in the literature review through empirical analysis, revealing the complex mechanism by which innovative teaching behaviors influence student learning engagement through the dual mediation of artificial intelligence trust and learning satisfaction. This study is among the first to model AI trust and learning satisfaction as parallel mediators between innovative teaching behaviors and learning engagement, thereby enriching our understanding of how pedagogical innovation and students’ psychological perceptions jointly shape engagement in the AI era.

The findings also provide concrete implications for educational practice. For teachers, they underscore the importance of adopting interactive, student-centered, and AI-supported teaching strategies, explicitly teaching students how to use AI critically (e.g., questioning outputs, checking sources), and continuously monitoring and enhancing students’ learning satisfaction through timely feedback, meaningful tasks, and supportive classroom climates. For institutions, the results point to the need to provide reliable AI infrastructure, sustained professional development on pedagogical uses of AI, and clear guidelines that encourage ethically grounded and pedagogically sound AI integration. For policymakers, the study highlights the value of developing standards and incentive structures that promote innovative teaching, safeguard data privacy and equity in AI use, and support systematic evaluation of how AI-enhanced teaching affects students’ trust, satisfaction, and engagement over time.

## Data Availability

The original contributions presented in the study are included in the article/Supplementary material, further inquiries can be directed to the corresponding author.
